# ErbB2/Her2-specific NK cells for adoptive immunotherapy of glioblastoma

**DOI:** 10.1186/2051-1426-2-S3-P41

**Published:** 2014-11-06

**Authors:** Congcong Zhang, Michael Burger, Lukas Jennewein, Sabrina Genßler, Manuel Grez, Torsten Tonn, Michel Mittelbronn, Joachim Steinbach, Winfried Wels

**Affiliations:** 1 Georg-Speyer-Haus, Institute for Tumor Biology and Experimental Therapy, Frankfurt am Main, Germany; 2 Institute for Neurooncology, Goethe University Frankfurt, Frankfurt am Main, Germany; 3 Edinger Institute, Goethe University Frankfurt, Frankfurt am Main, Germany; 4 German Red Cross Blood Service North-East, Dresden, Germany

## 

Significant progress has been made over the last decade towards realizing the potential of natural killer (NK) cells for cancer immunotherapy. NK cells can respond rapidly to transformed and stressed cells, and have the intrinsic potential to extravasate and reach their targets in almost all body tissues. In addition to donor-derived primary NK cells, also continuously expanding cytotoxic cell lines such as NK-92 are being considered for adoptive cancer immunotherapy. High cytotoxicity of NK-92 has previously been shown against malignant cells of hematologic origin in preclinical studies, and general safety of infusion of NK-92 cells has been established in Phase I clinical trials. To enhance their therapeutic utility, here we genetically modified NK-92 cells to express a chimeric antigen receptor (CAR), consisting of an ErbB2-specific scFv antibody fragment fused via a linker to a composite CD28-CD3 zeta signaling domain. GMP-compliant protocols for vector production, lentiviral transduction and expansion of a genetically modified NK-92 single cell clone (NK-92/5.28.z) were established. Functional analysis of NK-92/5.28.z cells revealed high and stable CAR expression, selective cytotoxicity against ErbB2-expressing but otherwise NK-resistant tumor cells of different origins in vitro, as well as homing to ErbB2-expressing tumors in vivo. Ongoing work now focuses on the development of these cells for adoptive immunotherapy of ErbB2-positive glioblastoma. We evaluated the activity of NK-92/5.28.z cells against a panel of glioblastoma cell lines and primary glioblastoma cultures and demonstrated selective in vitro cell killing that was dependent on the level of ErbB2 expression by the target cells and the time of their exposure to the NK cells. Antigen specificity and selective cytotoxicity of NK-92/5.28.z cells were retained in vivo, resulting in antitumoral activity against subcutaneous and orthotopic glioblastoma xenografts in NSG mice. Our results suggest adoptive transfer of ErbB2-specific NK-92/5.28.z cells as a promising new immunotherapy approach for ErbB2-positive glioblastoma.

